# Predictive and Prognostic Factors in Colorectal Cancer: A Personalized Approach

**DOI:** 10.3390/cancers3021622

**Published:** 2011-03-29

**Authors:** Myutan Kulendran, John F. Stebbing, Christopher G. Marks, Timothy A. Rockall

**Affiliations:** Department of Coloproctology, Royal Surrey County Hospital NHS Foundation Trust, Egerton Road, Guildford, UK

**Keywords:** predictive and prognostic factors, colorectal cancer, personalized medicine, APC, MSI, MMR, KRAS, BRAF, 18qLOH, CIMP, TGF-β, CIN, MGMT, TP53, FOBT, vimentin, circulating tumor cells

## Abstract

It is an exciting time for all those engaged in the treatment of colorectal cancer. The advent of new therapies presents the opportunity for a personalized approach to the patient. This approach considers the complex genetic mechanisms involved in tumorigenesis in addition to classical clinicopathological staging. The potential predictive and prognostic biomarkers which have stemmed from the study of the genetic basis of colorectal cancer and therapeutics are discussed with a focus on mismatch repair status, KRAS, BRAF, 18qLOH, CIMP and TGF-β.

## 1. Introduction

The idea of personalized medicine in colorectal cancer (CRC) is becoming a reality. Research into biomarkers in CRC lags behind other tumors, such as breast and lymphoma, in which gene testing results in the use of licensed medication [1]. Extensive investigation into metastatic disease has provided a better understanding of the genetic basis of tumorgenesis and more importantly identified the significance of the EGFR pathway and KRAS mutation [2-4]. The knowledge that patients with mutated KRAS do not respond to monoclonal antibody treatment in the metastatic setting has been an important step forward in attempting to tailor medication to the individual. This has led to careful genotyping of patients and identifying those with wild type-KRAS (WT-KRAS) for cetuximab and panitumumab therapy, leading to a reduction in chemotherapy toxicity and cost-effectiveness [5]. This finding has led to a host of other genetic biomarkers being studied in an aim to identify alterations that allow us to predict the prognosis of individual tumors and their response to therapy.

Clinicopathological staging determines treatment and plays a key role in selecting patients for clinical trials. The most robust determinants of prognosis include [6]:

- Local involvement (pT category of TNM);
- Regional lymph node metastasis (pN category of TNM staging);
- Lymphovascular invasion;
- Positive surgical margin;
- Pre-operative elevation of CEA;
- High tumor grade;
- Tumor budding.

In addition, patients who present with bowel perforation and obstruction are likely to have a poorer outcome because of locally advanced disease [7]. Although clinicopathological parameters determine the management of patients in the multi-disciplinary team setting they are not reliable predictors of treatment outcome. It is hoped the development of genetic biomarkers can be used in combination with clinicopathological staging. The current thinking behind CRC tumorgenesis and potential biomarkers that have been identified in the synthesis of this model will be discussed.

## 2. Adenoma-Carcinoma Sequence

**Figure 1.**
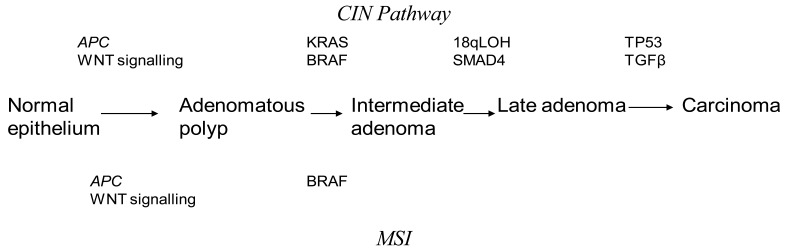
The modified Fearson and Volgenstein Model. Colorectal cancer (CRC) progression can occur through either the chromosomal instability (CIN) or microsatellite instability (MSI) pathway. Early adenomatous changes are secondary to loss of APC. KRAS loss initiates the formation of larger adenomas in the CIN pathway followed by 18qLOH. Mutations in TP53 are a late change. Sporadic MSI tumors are commonly part of the serrated neoplasia pathway and BRAF mutations are more common finding.

Discovery of genetic alterations in the pathogenesis of CRC are beginning to piece together how they may relate to the Fearson and Volgelstein Model [8]. Epigenetic alterations are thought to be precursor events in tumor progression through the serrated, alternate Vogelstein model [9]. It is believed that CRC may arise from at least three interlinked mechanisms. Chromosomal Instability (CIN) is the most commonly found in CRC accounting for up to 80% of cases [10]. The classic Vogelstein report, which describes the step by step mutational process starting from a small adenoma to invasive cancer, is the theoretical basis for our understanding of the CIN pathway [11]. CRC progresses through activating mutations in oncogenes or deactivation of tumor suppressor genes. This leads to a selection of clonal tumor cells which continue to divide through a growth advantage. Inactivating mutations in APC and activating mutations in KRAS are thought to be early changes in the Vogelstein sequence. Mutations in p53 and TGF-β have been described as late changes in tumorgenesis. An understanding of the CIN pathways has been pivotal in investigating potential predictive and prognostic markers in CRC. Unlike MSI tumors, the mechanism underlying CIN is poorly understood. CIN tumors are characterized by aneuploidy, multiple chromosomal rearrangements and an accumulation of somatic mutations [12] (Figure 1). CIN tumors have a poor prognosis compared to MSI tumors [10].

The loss of heterozygosity on the long arm of chromosome 18 (18qLOH) is the most common genetic alteration in colorectal cancer. SMAD4 and deleted in Colorectal Cancer (DCC) are two important tumor suppressor genes found on the long are of chromosome 18 [13,14]. Studies have shown that 18qLOH is an indicator of poor prognosis in early stage CRC, which has not been proven by multi-variate studies against other biomarkers, making 18qLOH an unlikely independent prognostic marker [15]. Furthermore 18qLOH has associations with CIN [16].

Deletion of SMAD4 by 18qLOH results in tumorgensis via the TGFβ pathway [17]. The loss of SMAD4 is a poor prognostic indicator. The retention of the SMAD4 diploidy results in a three-fold higher benefit from 5-Fluorouracil therapy (5-FU) chemotherapy [18].

Mutations in the tumor suppressor gene TP53 are found in almost half of CRC [19]. Mutations in different domains of the gene lead to a variable prognosis [20-24]. TP53 mutations are found more commonly in distal CRC [25,26]. Proximal tumors found to have mutations in TP53 were more likely to exhibit lymphatic invasion and be more responsive to 5-FU therapy. Mutation in exon 5 of the TP53 gene is associated with a poorer outcome [27]. Individuals with wild type TP53 have a superior survival rate with 5-FU therapy in rectal cancer [19]. At present there is no strong data to support the role of TP53 as a prognostic or predictive marker in CRC.

### 2.1. MSI

The most widely studied and understood genomic instability is Microsatellite Instability (MSI). MSI are found in 15% of CRC and is characterized by the inactivation of the Mismatch Repair Genes (MMR) [28,29]. MSI are the cause of hereditary CRC but are also found in sporadic cancers. In sporadic cases of MSI the MMR gene activity is silenced by promoter methylation of the hMLH1 gene [30,31]. Several genes affected by MSI have been identified including TGF-β [32], those encoding regulation of cell proliferation, cell cycle or apoptosis and DNA repair [33]. MSI represents a unique pathway for tumor development that does not involve loss of heterozygosity [34].

In Hereditary Non-Polyposis Coli Syndrome (HNPCC) a germline mutation in MMR occurs in an autosomal dominant fashion leading to MSI [30]. Although most research into MSI has focused on familial CRC only 3% of all CRC come from HNPCC and most MSI CRC are sporadic [35]. Macroscopically sporadic MSI tumors are characteristically proximally located, poorly differentiated and of a mucinous histology with lymphocytic infiltration [36]. The genetic properties of sporadic CRC include bialleleic methylation of the MLHI promoter, absence of MLH1 and PM2 protein and frequent mutations in BRAF [37].

MSI is a potential predictor of treatment response to 5-FU and prognosis of disease when used in conjunction with TNM staging [38]. Cultured CRC cells with intact MMR activity were more significantly sensitive to therapeutic concentration of 5-FU than DNA MMR deficient cells [39]. *In vitro* studies on CRC cells that do not express MLH1 has shown that exposure to the demethylating agent 5-azacytidine led to the expression of MLH1 and sensitivity to 5-FU [40].

MSI can be divided into MSI-High (MSI-H) tumors, which are MMR deficient, and MSI-Low (MSI-L) tumors, found to be proficient in MMR genes [41]. In the National Surgical Adjuvant Breast and Bowel Project (NSABP), CRC patients were divided into a non-treatment arm and an adjuvant chemotherapy arm and the MSI status was determined. The prognostic analyses showed increased recurrence-free survival (RFS) for MSI-H patients *versus* MSS/MSI-L patients (*P* = 10), but showed no difference in overall survival (OS; *P* = 67).

Retrospective studies on resected stage II and stage III CRC have shown that MSI-H tumors have a better prognosis compared to MSI-L tumors when not subjected to adjuvant chemotherapy [42]. *In vitro* studies have hypothesized that defective DNA MMR leads to 5-FU therapy resistance in CRC [43]. The Quick and Simple and Reliable Study (QUASAR) has shown that 5-FU therapy is beneficial in stage II CRC with the Overall Survival (OS) ranging between 1–5% [6]. In contrast the International Multi-centre Pooled Analysis of Colon Cancer Trials (IMPACT) B2 study did not show any advantage from combined 5-FU/leucovarin over surgery alone in Stage II CRC [44]. Furthermore 5-FU therapy may in fact be detrimental in this sub-group. Similar finding have been found in MSI-H, Stage III CRC *in vivo* [41]. There has been little evidence for the benefit of chemotherapy in patients with MSI CRC. The addition of topoisomerase-I inhibitor irinotecan in the chemotherapeutic regimen leads to increased survival times in patients with MSI tumors [45,46].

More recently, a new entity of MSI has been has been described. The signature is termed elevated microsatellite alterations at selected tetranucleotide repeats (EMAST). EMAST can be found in up to 60% of sporadic CRC and co-exists with MSI-H and MSI-L tumors. EMAST is thought to occur due to down regulation of MSH-3 [47].

It can be argued that MSI status should be routinely determined as part of staging CRC. At present there is no general consensus for deciding on who should receive adjuvant chemotherapy in stage II disease. It is unclear if all patients with stage II disease will benefit from adjuvant chemotherapy, MSI status may be a candidate for determining suitability for adjuvant chemotherapy.

The addition of oxaliplatin with infusional 5-FU in FOLFOX therapy has demonstrated a significant improvement in three-year disease free survival in patients with stage II, node negative disease. There is currently no published data with regard to the interaction between oxaliplatin and MSI status. The prognostic significance of MSI is unquestionable but it is essential to know more about CRC genetic status than MSI alone, these potential biomarkers shall be discussed below.

### 2.2. KRAS

Kirsten-ras (KRAS) mutations are the most widely studied and promising biomarker for treatment strategies in CRC. It is a downstream mediator of the EGFR signaling pathway [16]. KRAS mutations are an early event in the adenoma-carcinoma sequence and are found in 40% of CRC [8]. KRAS mutations are thought to be a poor prognostic marker in CRC [48]. There is, however, conflicting evidence. The KRAS in-colorectal-cancer collaborative group (RASCAL II) study has shown that a glycine to valine mutation on codon 12 of the KRAS gene is aggressive in patients with Duke's C CRC [49]. It was associated with a 50% increased risk of relapse or death in this group of patients.

KRAS mutations are maintained in metastatic CRC [50,51]. Mutant KRAS exhibits resistance to monoclonal antibody (mAb) therapy [3,4]. This phenomenon has been studied extensively in metastatic disease. Randomized controlled trials have shown no survival benefit from adding cetuximab to chemotherapy in unselected patients [52-54]. The large, randomized, phase II Oxaliplatin and Cetuximab in First-Line Treatment of CRC (OPUS) trial has indicated that it may be harmful to add cetuximab to those with mutated KRAS [55]. A Phase III trial (the CRYSTAL study) confirms that a combination of cetuximab and FOLFIRI statistically improves response rate and PFS in the WT-KRAS population [52]. Current practice involves genotyping patients with metastatic CRC prior to starting mAb therapy. The European Health Committee for human medicinal products has recommended the use of panitumumab monotherapy and cetuximab therapy in patients with metastatic CRC found to have WT-KRAS in the primary tumor [56].

PIK3CA is a downstream effector of the KRAS pathway and is down regulated by the tumor-suppressor gene PTEN. PIK3CA is a potential marker of resistance to mAb therapy [57].

### 2.3. BRAF

BRAF mutations are found in 10% of CRC. Most B-Raf mutations involve the V600E amino acid substitution, resulting in constitutive activation of the MEK-ERK signaling pathway. They are mutually exclusive of KRAS mutations and are thus a candidate for an independent biomarker for CRC [58]. Studies on WT-KRAS tumors treated by either a combination of chemotherapy and cetuximab or cetuximab alone have shown that progression free survival (PFS) and OS were significantly better among patient without mutated BRAF (WT-BRAF) [59]. The presence of BRAF mutations in an MSI tumor makes a hereditary cause unlikely [60,61]. Therefore the strategy for identifying individuals with Lynch syndrome is a two tier approach. The initial test involves immunohistochemistry testing for MMR protein in tissue and MSI DNA testing. Individuals found to be MSI^+^ with a loss of MMR proteins (MSH2, MHS6 and PMS2) undergo further DNA testing for the appropriate gene. Patients who are MSI^+^ with either a loss of MLH1 or no MMR protein loss undergo Tier 2 screening. This consists of BRAF mutational analysis at V600E and MLH1 promoter methylation, both of which are associated with sporadic CRC [62,63].

At present, inhibitors of BRAF have been tested *in vitro* and the inhibitor PLX4032 has been shown to potentiate the anti-proliferative action of 5-FU therapy [64]. At present, further *in vivo* studies investigating the action of the tyrosine kinase inhibitor (BAY 43-9006) and cetuximab in the metastatic setting are being undertaken. Tyrosine kinase inhibitors are believed to restore sensitivity to mAb therapy in mutant-BRAF cell lines [4]. They show promise as potential treatment methods. However deliverability and costs of treatment are yet to be evaluated.

### 2.4. CIMP

DNA methylation is recognized as one of the most common gene alterations in human tumors including CRC [65]. A subset of CRC exhibit promoter methylation at multiple sites and are referred to as the CpG island methylator phenotype (CIMP) [66,67]. The CIMP is observed in 30% of CRC. This has been hypothesis as an early contributor to CRC progression [68]. Both hyper and hypo-methylation of DNA play a role in CRC tumorogenesis [69]. Before the entity of CIMP was identified, CRC was classified into either MSI or CIN in origin. It now apparent that come tumors are neither MSI or CIN and that hypermethylation of DNA is a common finding in CRC [70,71]. Sporadic MSI tumors are secondary to CIMP related silencing of the MMR gene MLH1 [63,72]. The difficulty producing a standardized marker and the unclear distinction between the CIMP tumors and sporadic MSI tumors has meant that the clinical importance of CIMP tumors is difficult to quantify. CIMP can be divided into CIMP-High (CIMP-H) and CIMP-Low (CIMP-L) groups [70]. The CIMP-H tumors are associated with the BRAF mutation whereas CIMP-L are associated with KRAS mutations [73-75]. CIMP-H tumors found to be MSI positive and haboring the BRAF mutation have a good prognosis [59]. However MSI negative tumors which are positive for CIMP and the BRAF mutation hold a poor prognosis [76]. Global hypomethylation of DNA is associated with CIN tumors and may also confer a poor prognosis [77,78].

*In vitro* it is possible to demethylate some promoters using the DNA methyltransferase inhibitor 5-azacytidine although remethylation occurs on removal of the agent. The search for a targeted and more prolonged acting agent may be of therapeutic use [79].

Gene expression arrays comparing CRC tissue with adjacent normal mucosa are identifying and validating novel methylated genes that may in the future be candidate prognostic biomarkers [80-85]. Aberrant DNA methylation can be detected from a variety of samples including blood, stool and tissue, giving it a wide variety of clinical uses [86-88].

CIMP positivity in CRC was thought to be a significant independent predictor of survival benefit from 5-FU chemotherapy [89]. However, recent studies have failed to prove this for Stage II-III CRC [90].

It would be ideal to combine the molecular classification of CRC with DNA methylation status to predict treatment response and prognosis. The role of epigenetic therapy has been proven to be effective in treating hematological malignancies and is likely to extend to CRC in the future [91].

### 2.5. MGMT

The DNA repair gene 06-methylguanine-DNA methyletransferase (MGMT) is often methylated in CRC [92]. Sporadic cancer is thought to arise from regions of cells with field change. MGMT has been detected in healthy tissue surrounding tumor cells [93]. It has been hypothesized that MGMT field change may represent a preneoplastic state for the development of MSI tumors. In support of this there is an increase in promoter DNA methylation as tumors progress through the adenoma sequence with increasing malignant potential. MGMT may serve as a prognostic marker for CRC, however recent immunohistochemistry studies have failed to show any association of MGMT promoter methylation or loss as a prognostic biomarker in CRC [94].

### 2.6. Early Detection of CRC

The most evaluated screening tool for the detection of CRC is the biennel Faecal Occult Blood Test (FOBT). The sensitivity of the FOBT for important neoplasms remains low, between 30–50% [95]. Those who test positive are invited for colonoscopy which detects and allows excision of adenomas reducing the incidence of cancers by 16% [96,97]. The early detection of cancers allows for treatment of less advanced disease. An alternative to FOBT would be fecal DNA based tests. Such tests that detect DNA mutations are complicated, expensive and also sensitive to adenomas [98].

In the USA, stool based methylated Vimentin (m-Vimentib) is currently available commercially. The vimentin gene, which is transcriptionally silent in normal colorectal epithelial cells, becomes methylated in colorectal cancers [99]. The development of more sensitive technology enables absolute quantification of the number of methylated molecules in a sample, and detection of m-Vimentin in plasma may act as a potential biomarker or tumor marker [100].

Circulating tumor cells is FDA approved for patient prognosis in metastatic colorectal cancer [101-103] and may in the future act as a predictive biomarker as well as give an indication of cell dissemination during surgery [104].

## 3. Discussion

New evidence highlighting the variation in altered pathways leading to CRC has provided a modified version of the classic Vogelstein and Fearson model. At present it is thought that there are three parallel pathways which give rise to sporadic colorectal cancer with distinct clinicopathological features.

MSI tumors are associated with the serrated neoplasia pathway and frequently carry the BRAF mutation [105]. These tumors are often CIMP positive. CIN tumors are activated by biallelic loss of APC and p53 mutation classically forming tubular adenomas of the distal colon. The CIMP pathway is heterogenous in nature; again there is a strong association with the BRAF mutation. The prognostic value of promoter hypermethylation is still under investigation. The role of BRAF mutation in V600E and MSI already play a role in determining a hereditary cause for CRC, their usefulness in sporadic CRC is now beginning to merge.

Identification of KRAS and subsequent testing for this marker has opened the doors for personalized medicine and further research into other potential biomarkers. MAb therapy targeting the EGFR pathway has shown great efficacy in the treatment of patients with metastatic CRC. However, anti-EGFR therapy is only useful for a fraction of patients, making it essential to look for alternative pathways and inhibitors. There is a vast array of new targets being studied as possible new biomarkers, some of which are being tested clinically to patients.

The commercially available OncoTypeDx is a RT-PCR gene assay, which detects 12 validated genes in CRC and produces a recurrence score for Stage II and II disease after resection. The assay is not predictive of treatment response. The seven prognostic genes include three stromal: FAP, INHBA and BGN; three cell type genes: Ki-67, c-myc and MYBL2 and GADD45B [106].

The evolution of CRC progression from an adenomatous polyp to invasive cancer and metastasis may be dependent on protein markers [107]. Among those studied, cell surface markers show promise for further development. Cell surface markers are necessary for cell-cell adhesion and communication. The role of stroma-derived biomarkers, tumor-associated macrophages, infiltrating lymphocytes and small transmembrane proteins have been described [108]. Despite the discovery of a wide range of protein biomarkers for CRC, the translation into clinical practice is challenging due to the difficulty in detecting and characterizing low abundance proteins in complex mixtures and the validation of biomarkers in clinical practice. Issues with regard to validity arise from the multitude of marker assessment methods, feasibility of obtaining the specimens, reliability and reproducibility of the assay and the costs involved with assessing the marker status on every patient [109].

An ideal way to assess tumor status would be a serum marker. Serum markers allow for a minimally invasive method of CRC screening that could easily integrated into regular health checks. Six serum biomarkers have been studied using ELISA. CEA showed the best sensitivity at 95%, with a specificity of 43.9% followed by seprase (42.4% sensitivity), CYFRA 21-1 (35.5%), osteopntin (30.2%), ferritin (23.9%) and anti-p53 (20.0%). When used in combination the sensitivity of these markers was equal to fecal immunohistochemical testing [110].

Other novel markers for further investigation include Single-Nucleotide Polymorphisms (SNPs). The study of SNPs has identified variants in SMAD7 associated with CRC. The SNPs rs4939827, rs1295317 and rs4464148 have shown evidence of an association between genotype and risk in three independent CRC case-controlled series by allelic-specific PCR. The locus on SMAD 7 may contribute up to 15% of CRC [111].

## 4. Conclusions

The revolution of personalized medicine not only benefits the patient by reducing drug toxicity and optimizing patient outcome but can also reduce costs for an already burdened health system. In the future, personalized medicine means that therapeutic regimens will be tailored more and more to the individual. The era of personalized medicine opens a very exciting time for the management of colorectal cancer and in combination with minimally invasive surgical techniques will benefit the patient greatly.

## Glossary of Abbreviations

CEA: Carcinoembryonic antigen, tumor marker
Cetuximab: Chimeric monoclonal antibody, Epidermal growth factor receptor inhibitor
CRC: Colorectal cancer
CIMP: CpG Island methylator phenotype, DNA hypermethylation characterized by epigenetic instability
CIN: Chromosomal Instability, Mutation of CIN genes increases the probability that whole chromosomes or large fractions of chromosomes are gained or lost during cell division
EGFR: Epidermal Growth Factor Receptor, cell surface receptor
KRAS: Kirsten-ras, oncogene
MMR: DNA Mismatch Repair system, functions during replication, Mismatch repair protein include: MLH1, MSH2, MSH6 and PMS2
MSI: Microsatellite Instability, secondary to defect in normal DNA repair process, Resulting in variable microsatellite lengths
MSS: Microsatellite stable tumors
Pantuximab: Chimeric monoclonal antibody, Epidermal growth factor receptor inhibitor
TNM: Tumor, Node, Metastasis, cancer staging system
WT-KRAS: Wild Type KRAS, non-mutated form
SNP: Single-nucleotide polymorphisms
